# Prediction and Optimization of Load-Bearing Capacity in Resistance Spot Welded Titanium Joints Using Neural Networks and Genetic Algorithms

**DOI:** 10.3390/ma19112184

**Published:** 2026-05-22

**Authors:** Piotr Lacki, Wojciech Więckowski, Michał Lacki, Marcin Dyner, Janina Adamus

**Affiliations:** 1 Faculty of Civil Engineering, Czestochowa University of Technology, J.H. Dabrowskiego 69 Str., 42-201 Częstochowa, Poland; piotr.lacki@pcz.pl; 2 Faculty of Mechanical Engineering, Czestochowa University of Technology, J.H. Dabrowskiego 69 Str., 42-201 Częstochowa, Poland; wojciech.wieckowski@pcz.pl; 3 Faculty of Computer Science and Artificial Intelligence, Czestochowa University of Technology, J.H. Dabrowskiego 69 Str., 42-201 Czestochowa, Poland; michal.lacki3210@gmail.com; 4 Faculty of Science and Technology, Jan Dlugosz University in Czestochowa, 13/15 Armii Krajowej Ave., 42-200 Czestochowa, Poland; m.dyner@ujd.edu.pl

**Keywords:** resistance spot welding, titanium alloys, artificial neural networks, genetic algorithm, load bearing capacity, process optimization, welding parameters, dissimilar joints

## Abstract

This study investigates the mechanical performance of resistance spot-welded titanium lap joints made of Grade 1 and Grade 5 alloys. Experimental tests were combined with artificial neural network modeling to predict joint load-bearing capacity based on welding current and welding time. Three models were developed for Grade 1/Grade 1, Grade 1/Grade 5, and Grade 5/Grade 5 joints. The mixed Grade 1/Grade 5 joint achieved the highest predictive accuracy, with an R^2^ value of 0.9289. Statistical evaluation confirmed high model reliability, with mean relative errors between four and six percent. The most accurate model was optimized using a genetic algorithm. The algorithm identified an optimal parameter set consisting of a welding current of 2.89 kA and a welding time of five pulses. This configuration produced a predicted load-bearing capacity of 3.2 kN, which meets the required threshold of three kilonewtons. Contour maps showed that the optimal point lies near the boundary of the high-strength region and corresponds to the lowest welding current and shortest welding time that still ensure sufficient joint quality. The results demonstrate that combining neural network modeling with evolutionary optimization is an effective approach for designing efficient welding processes for dissimilar titanium joints.

## 1. Introduction

Resistance spot welding (RSW) is the dominant joining technique in the automotive sector, valued for its low cost, high reliability, and straightforward integration into high-volume production lines. It is widely used for joining thin-walled metallic components in the automotive, aerospace, electronics, and household appliance sectors. The process relies on localized heating caused by the flow of electric current through electrodes under simultaneous mechanical pressure. Electrical resistance at the interface between the joined components leads to localized melting and the formation of a permanent joint. Despite its apparent simplicity, the RSW process is complex and highly sensitive to a range of technological parameters, including:

- Electrode force during welding, which ensures proper contact between the components and enables current flow;
- Welding time;
- Current intensity at the weld point;
- Electrode material and geometry;
- Physical and mechanical properties of the base materials.

In recent decades, RSW has been widely applied to the joining of low-carbon steels, Advanced High-Strength Steels (AHSSs), and aluminum alloys. Increasing attention has been directed toward non-destructive evaluation of weld quality without compromising the physical or mechanical integrity of the joint. Early studies, such as the work by Chen et al. [1], demonstrated that real-time acquisition and analysis of welding parameters can provide reliable information for assessing the quality of spot-welded titanium alloys. Their approach, based on extracting characteristic signal features during the welding cycle, highlighted the potential of online monitoring systems to improve process stability and weld integrity. Building on these foundations, subsequent research has increasingly focused on predictive and optimization techniques that support real-time quality assessment and process control, including classical statistical approaches (e.g., regression analysis, Taguchi method) as well as advanced Artificial Intelligence (AI) methods such as supervised and unsupervised machine learning, artificial neural networks (ANNs), deep neural networks (DNNs), convolutional neural networks (CNNs), recurrent neural net-works (RNNs), reinforcement learning (RL), support vector machines (SVMs), decision trees, ensemble methods such as random forests (RFs) and gradient boosting (GB), and optimization algorithms like genetic algorithms (GAs) and particle swarm optimization (PSO). For example, Chuenmee et al. [2] implemented five machine learning algorithms—ANNs, CNNs, LSTM, Random Forest Classifier (RFCs), and Extreme Gradient Boosting (XGBoost)—to predict weld quality and enable full process control of RSW in automotive body manufacturing. Similarly, Domínguez-Molina et al. [3] developed a non-destructive weld quality assessment method using computer vision models. Based on process input parameters (current, time, electrode force) and both visible and thermal images, they trained six machine learning models capable of classifying weld quality. Their findings demonstrated that welding time and electrode angle significantly affect mechanical strength, and that visible images yield higher prediction accuracy than thermal ones.

Despite the growing importance of titanium and its alloys in aerospace, automotive, marine, energy, and medical applications [4,5,6,7], their use in RSW remains significantly less explored. Titanium is characterized by low thermal conductivity, high chemical reactivity at welding temperatures, and a tendency to form brittle intermetallic phases in dissimilar metal joints [8]. Additionally, recent findings by Motyka et al. [9] showed that even moderate thermal exposure can induce pronounced diffusion-driven microstructural changes at α/β interfaces, underscoring the material’s sensitivity to thermal cycles. These properties make the RSW process for titanium particularly demanding, requiring precise control of process parameters. The sensitivity of RSW to even small fluctuations in welding current has also been confirmed in recent studies. Mikno and Stępień [10] demonstrated that power losses and voltage drops occurring in the supply system of resistance welding machines can significantly reduce the effective welding current, leading to a noticeable decrease in nugget diameter and joint strength. Their findings further emphasize the need for precise control of process parameters, particularly in materials as demanding as titanium. Gandhi and Solanki [11], in a comprehensive review of RSW advancements for titanium and its alloys, identified sheet thickness and electrode diameter as key technological parameters influencing weld quality. They highlighted notable research gaps regarding the influence of these factors and emphasized the need for further investigation into specific grades such as CP Grade 2. The authors also pointed out that thin-walled joints require special attention due to challenges in process control, nugget stability, and the risk of structural defects. They stressed the importance of optimizing RSW parameters for varying material thicknesses and electrode geometries, which is critical for industrial applications.

In dissimilar metal joints such as titanium–steel, the use of interlayers (e.g., nickel, nickel–copper, niobium) is essential to suppress the formation of brittle intermetallic phases and enhance joint quality. Studies by Liu et al. [12], Wang et al. [13], and Zhang et al. [14] confirm the effectiveness of such approaches, demonstrating improvements in both mechanical and microstructural properties of Ti/Steel welds. The review by Liu and Zhang [15] showed that interlayers such as Cu, Ni, Nb, and Ni–Cu alloys can effectively limit the diffusion of Fe and Ti and promote the formation of intermetallic compounds with lower brittleness, thereby reducing internal stress and improving joint strength. Furthermore, research by Jiang Yu et al. [16] revealed that applying a Ni–Cu interlayer in the RSW process between Q235 steel and Ti6Al4V alloy led to the formation of a reaction and diffusion layer, replacing the conventional weld nugget. The resulting joints exhibited more than twice the shear strength and showed no structural defects compared to those produced without an interlayer. The use of interlayers becomes particularly critical in thin-walled components, where even minor microstructural imperfections may lead to premature failure or loss of functionality.

In applications where weld quality is critical to user safety and product durability, it is essential to develop methods that enable precise control of the welding process and real-time evaluation of joint integrity.

The application of AI in optimizing the RSW process for titanium is a promising research direction. Mezher et al. [17,18,19,20] conducted comprehensive studies on resistance spot welding of Grade 2 titanium sheets and AISI 304 austenitic stainless steel, focusing on mechanical, structural, and predictive aspects. A series of experimental trials evaluated the influence of process parameters, such as welding current, electrode force, welding time, pulsed welding, and material thickness, on joint quality, measured by shear strength, nugget diameter, microhardness, and fatigue life. Advanced machine learning models, including ANN, CatBoost, and Random Forest, were trained on data from over 100 welding trials. The best predictions were achieved using an ANN model with Levenberg–Marquardt training and a log-sigmoid transfer function, reaching a coefficient of determination R^2^ = 0.984 and mean squared error MSE = 0.018. In terms of fatigue performance, joints with equal sheet thicknesses (e.g., 0.5–0.5 mm) exhibited higher strength and longer service life compared to configurations with unequal thicknesses (e.g., 1.0–0.5 mm). However, certain combinations of dissimilar thicknesses also demonstrated favorable fatigue properties when process parameters were properly adjusted. SEM analysis revealed differences in fracture mechanisms, with ductile failure occurring in similar joints and brittle fracture in dissimilar ones, which are characterized by distinct features on the fracture surfaces. The authors emphasize the importance of precise control over RSW parameters and highlight the potential of AI-based methods for optimizing the joining of hard-to-weld materials, particularly in aerospace and automotive applications.

Additionally, Zhao et al. [21] and Butsykin et al. [22] proposed advanced methods for monitoring the resistance spot welding process based on power signal analysis and dynamic electrical resistance. Zhao et al. [21] compared the effectiveness of stepwise regression and ANN in predicting nugget diameter using power signal characteristics derived from current and voltage measurements during the RSW of TC2 titanium alloy. In turn, Butsykin et al. [22] evaluated the reliability of process control by monitoring the dynamic resistance between electrodes (RBE), demonstrating that the use of low-current pre-pulses and feedback from digital power sources enables thermal stabilization and improves weld quality. Both approaches support the development of adaptive systems and real-time non-destructive quality monitoring, particularly in the context of hard-to-weld materials such as titanium and its alloys.

Zhao et al. [23] proposed an entropy weight method combined with regression analysis to optimize the resistance spot welding process. Their model enables simultaneous improvement of weld quality and reduction of excessive heat input, identifying welding current and welding time as the most influential parameters affecting joint performance. The approach can serve as a practical guideline for RSW operators to ensure weld integrity while meeting the demands of high-volume production and stable process control.

In the context of Industry 4.0, the integration of AI methods with manufacturing systems enables not only the automation of the welding process but also its continuous optimization based on sensor data. This allows for dynamic adjustment of process parameters in response to changing material and environmental conditions, resulting in improved efficiency, repeatability, and reliability. For hard-to-weld materials such as titanium, the benefits of AI-driven process control are particularly evident.

Despite the growing number of publications on the application of AI in welding, there is a lack of systematic research on the resistance spot welding of thin titanium sheets, particularly in the context of process optimization using artificial intelligence methods. Most available studies focus on commonly used and more easily weldable materials such as steel or aluminum, or on joining titanium components with thicker cross-sections. In contrast, issues related to welding thin titanium sheets (<1 mm) are addressed less frequently, due to technological challenges and limited availability of experimental data. Meanwhile, thin-walled titanium components are gaining importance in certain structural applications, which justifies the need for an in-depth analysis of the RSW process in this area.

The novelty of this study lies in the combined experimental and predictive analysis of resistance spot-welded joints made from commercially pure titanium Grade 1 and titanium alloy Grade 5 (Ti-6Al-4V), joined in three configurations: Grade 1/Grade 1 (G1/G1), Grade 5/Grade 5 (G5/G5) and Grade 1/Grade 5 (G1/G5). All joints were produced using thin sheets with a thickness of 0.8 mm, a configuration that remains insufficiently documented in the literature, particularly in the context of process optimization for thin-walled titanium components. To address this gap, the study introduces three artificial neural network models developed entirely by the authors to predict the load-bearing capacity of the joints based on welding current and welding time. This focused integration of experimental work and AI-based prediction provides new insights into the behaviour of RSW joints in technologically sensitive titanium systems that require precise control of process parameters.

## 2. Materials and Methods

**Figure 1:**
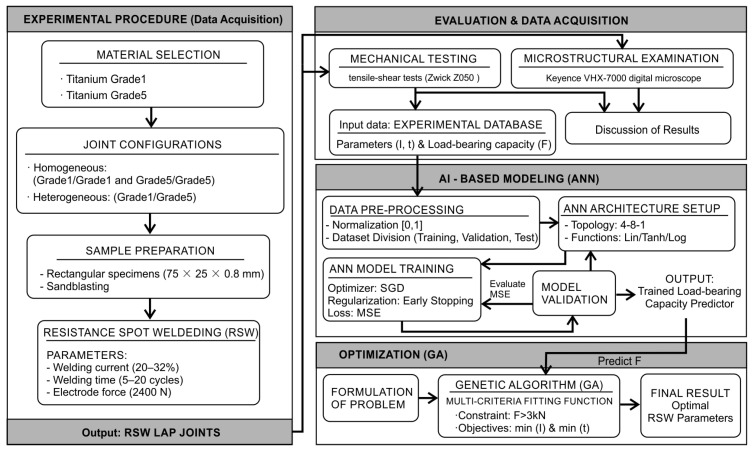
Flowchart illustrating the algorithm used in the study.

The present study employs an integrated research methodology that links experimental investigations of the Resistance Spot Welding (RSW) process with advanced computational tools. The overall workflow, encompassing data acquisition, Artificial Neural Network (ANN) modeling, and multi-criteria Genetic Algorithm (GA) optimization, is illustrated in Figure 1.

### 2.1. Materials

The experimental work was carried out on resistance spot-welded (RSW) joints made from commercially pure titanium Grade 1 and titanium alloy Grade 5 (Ti-6Al-4V). Three configurations of lap joints were produced: homogeneous (Grade 1/Grade 1 and Grade 5/Grade 5) and heterogeneous (Grade 1/Grade 5). The chemical compositions of the investigated materials are presented in Table 1 and Table 2.

The mechanical properties of the sheets are summarized in Table 3.

### 2.2. Sample Preparation and Welding Procedure

**Figure 2:**
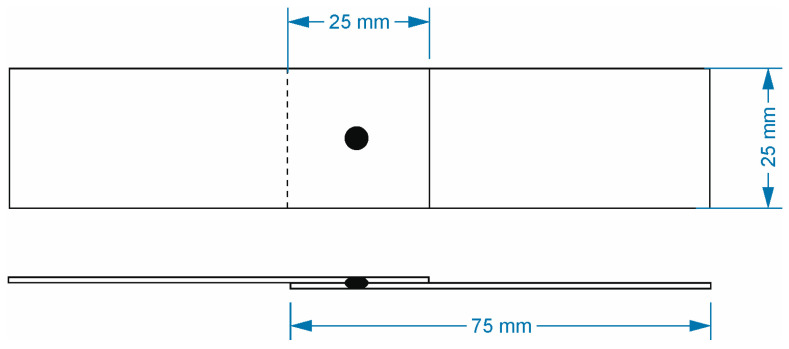
Geometry of the RSW lap joint.

Rectangular specimens with dimensions of 75 × 25 mm were cut from 0.8 mm-thick sheets. The geometry of the lap joint configuration is shown in Figure 2.

Prior to welding, the faying surfaces were sandblasted to remove contaminants and oxide layers, ensuring stable electrical contact and repeatable thermal conditions during welding.

**Figure 3:**
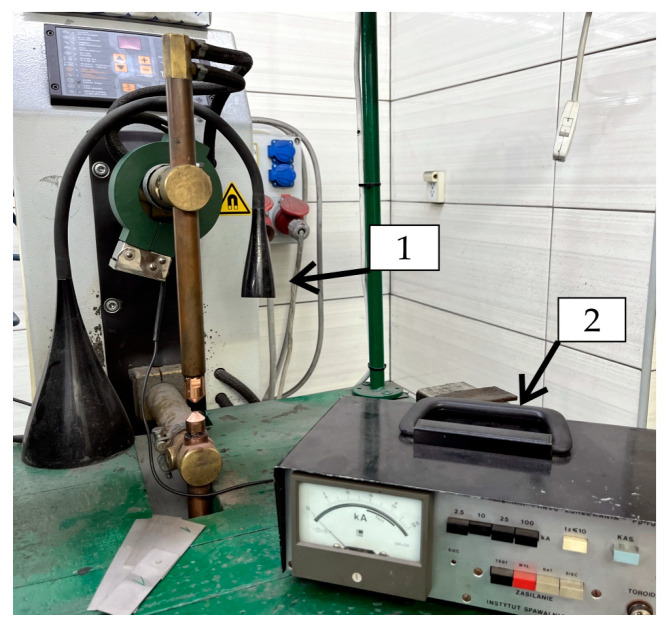
Experimental setup: (1) TECNA 4640 spot welding machine, (2) Pp-7d welding current meter.

All welding experiments were performed at the Manufacturer of Surgical Instruments CHIRMED (Rudniki near Częstochowa, Poland) using a TECNA 4640 resistance spot welding machine (VIA GRIECO 25/27–400024, Castel S. Pietro, Bologna, Italy). The welding process was carried out using alternating current (AC). The system was equipped with Cu electrodes with a tip diameter of 3.5 mm. The working surface of the electrode is flat and circular, corresponding to an active area of approximately 9.6 mm^2^. The experimental setup is shown in Figure 3.

The technical specifications of the welding machine are listed in Table 4.

The welding process was carried out under industrial conditions using various combinations of welding current settings and welding times, while maintaining a constant electrode force. For each joint configuration (Grade 1/Grade 1, Grade 5/Grade 5, Grade 1/Grade 5), sixteen welding parameter sets were applied, as summarized in Table 5. For every parameter set, four specimens were prepared: three for tensile-shear testing (48 specimens in total) and one for microstructural examination (16 specimens in total), resulting in 64 specimens per joint configuration.

The actual welding current (kA) was measured using a Pp-7d current meter (Institute of Welding, Gliwice, Poland), ensuring accurate verification of the electrical parameters during each welding cycle. The device records the RMS (effective) value of the welding current, the standard parameter used to characterize resistance spot welding processes. Its basic measurement error of ±2.5% of the measured value.

According to the technical specifications, the minimum measurement time of the Pp-7d meter is 60 ms for welding times ≤ 10 cycles and 200 ms for welding times > 10 cycles. The basic measurement error of ±2.5% applies uniformly to all welding times used in this study, including 0.1 s and 0.4 s.

### 2.3. Mechanical and Microstructural Evaluation

Mechanical and metallographic tests were carried out under laboratory conditions using the specimens prepared as described in Section 2.2.

Tensile-shear tests were performed using a Zwick Z050 universal testing machine (ZwickRoell GmbH & Co. KG, Ulm, Germany) at a constant crosshead speed of 2 mm/min.

Specimens intended for metallographic analysis were sectioned along the weld axis, followed by standard grinding, polishing, and etching procedures. Microstructural observations were conducted using a Keyence VHX-7000 digital microscope (KEYENCE Corporation, Osaka, Japan).

### 2.4. Research Methodology for Two-Level Factorial Design (2^k^)

To assess the effect of process parameters on joint load-bearing capacity, a Design of Experiments (DOE) methodology based on two-level factorial designs was employed. A 2(2-0) model was used, this means examining two independent variables at two levels of variability (low and high), with a value of 0 indicating no fractionalization of the design. This achieved full resolution (Resolution = FULL), which in research practice ensures that main effects and interactions between variables are not confounded (aliased) and can be estimated independently and unambiguously. The experimental structure was designed to ensure high statistical reliability:

- Experimental Matrix. A standard two-factorial design includes four basic experimental setups (corner points), representing all possible combinations of factor levels.
- Replications and Center Points. The total number of runs was expanded to 30. A key element of the methodology was the introduction of 18 (1 + 17) center points. Repeating the measurement multiple times at the geometric center of the plan allows for extremely precise determination of the Pure Error and verification of the model’s linearity.
- Process stability. The use of varying the number of replications (2–17) for individual plan points and conducting the tests within a single block (Number of blocks: 1) minimized the impact of confounding variables and instrument drift.

The Full Factorial methodology used provides the foundation for statistical analysis, enabling not only the identification of factors with a statistically significant effect on the dependent variable (DV) but also the precise definition of the mathematical regression equation describing the phenomenon being studied. This research plan ensures an optimal balance between the number of tests performed and the quality of the information obtained about the process.

### 2.5. Research Methodology for Central Composite Design (CCD) and Response Surface Method (RSM)

To comprehensively optimize the RSW process parameters and determine their impact on the joint’s load-bearing capacity, a DOE methodology was employed. CCD, a non-factorial surface design designed to model nonlinear relationships, was applied. This method was chosen because it was necessary to go beyond simple linear relation-ships and develop a mathematical Response Surface Model (RSM), which allows for the precise determination of optimal points for the studied process. The experimental structure is based on the following design assumptions:

- Independent Variables. The model includes two factors, which, using the CCD, allows for the generation of a quadratic transfer function.
- Sample Size and Replications. The total number of measurements was 53 for G1/G1, 52 for G1/G5, and 54 for G5/G5 experiments (runs). The studies were conducted across 16 unique experimental designs, meaning that individual points in the design were repeated (replicated) two to four times. This high number of repetitions is crucial for reliable estimation of the pure error and model stability.
- Design characteristics. A single-block design was used, ensuring that all tests were performed under uniform environmental conditions, minimizing the risk of bias.
- Response Surface Analysis. By extending the classic factorial design with star points and multiple center points, it was possible to generate a three-dimensional mathematical model. This model allows visualization of the impact of interactions between factors and identification of regions where the dependent variable (DV—joint load-bearing capacity) takes on extreme values.

The methodology used transforms a set of point-based measurement data into a continuous mathematical model. This approach enables the prediction of the load capacity of welded joints, even for parameter combinations that were not directly tested in the laboratory.

### 2.6. AI-Based Modelling Procedure

Three artificial neural network models were developed as part of the study to predict the load-bearing capacity of joints depending on resistance welding process parameters: current intensity and welding time. The models were differentiated by the type of materials being joined, analyzing the following configurations: Grade 1/Grade 1, Grade 5/Grade 5, Grade 1/Grade 5.

The artificial neural network (ANN) and genetic algorithm (GA) used in this study were implemented using proprietary software developed specifically for the purposes of this research. The computational framework was created by the authors and is not based on any commercial or publicly available software packages.

#### 2.6.1. Data Preparation

Separate datasets were generated for each welding joint configuration. To ensure numerical stability and optimize the training process, all collected experimental data were normalized to the range [0, 1]. The dataset construction strategy also included records of repeated tests for the same process conditions, which allowed the networks to average results and increase the models’ robustness to measurement errors. These prepared databases were divided into training, validation, and test subsets in proportions that ensured a reliable assessment of the network’s generalization capabilities. The detailed record counts and their division into training, test, and validation subsets are presented in Table 6. The data were divided by the type of joints subjected to resistance spot welding. This division was designed to provide the models with an adequate number of examples for training while maintaining representative data groups for an objective assessment of their predictive capabilities.

The training subset was intentionally the largest to provide the ANN with a sufficient number of examples for effective learning, especially given the relatively small size of the experimental datasets. The validation and test subsets were selected to maintain a balance between preventing overfitting and enabling an unbiased evaluation of model performance. Minor differences in the percentage distribution between joint configurations result from the total number of available samples.

It should also be noted that the number of records listed in Table 6 exceeds the 48 specimens produced for each joint configuration during the main test series. This is be-cause additional preliminary welds were prepared to establish the appropriate welding parameter ranges. These preliminary joints were included in the ANN training dataset, resulting in a total of 53 samples for G1/G1, 52 for G1/G5, and 54 for G5/G5.

The input structure of the models included four variables: the set current intensity in percent, the actual measured current intensity in kA, and the welding time expressed in both cycles and seconds. The output data used to verify the model’s effectiveness was the measured joint load-bearing capacity expressed in kilonewtons (kN).

The selection of four input parameters for the artificial neural network (ANN), despite their mutual correlation, is a deliberate methodological choice aimed at improving the stability and predictive precision of the model. The data-collection strategy, which included repeated tests under identical conditions, was designed to average the results and increase the network’s robustness to informational noise. The redundant inputs, combined with the stochastic gradient descent (SGD) method and the optimized network topology (4-8-1), enable more effective extraction of nonlinear features from the input data.

Although the set current expressed in percent (%) and the measured current in kiloamperes (kA) are strongly correlated, they convey different information to the neural network. The set current (%) is a discrete machine-control parameter (e.g., 20, 24, 28, 32%) representing the operator’s intended setting. The measured current (kA) is a continuous variable reflecting the actual electrical response of the welding system, recorded by an external Pp-7d meter. Under industrial conditions, the same percentage setting may result in slightly different current values due to fluctuations in supply voltage, electrode wear, or changes in contact resistance. Including both parameters allows the network to “see” these physical fluctuations, increasing its robustness to measurement errors and process variability.

From a mathematical perspective, the information on welding time expressed in cycles (where 1 cycle = 20 ms) and in seconds is equivalent. However, in the ANN architecture all input data undergo normalization. Providing two representations of the same physical quantity (time) supports stabilization of the learning process in the input layer, which consists of four neurons. Time in cycles is the standard unit used in welding machine settings (based on the 50 Hz mains frequency), whereas seconds are the physical unit used in numerical modelling. Including both forms ensures completeness of the data in a process where precise time control is critical for weld nugget quality.

Although welding time expressed in cycles and in seconds represents the same physical quantity, the two values differ numerically after normalization and, therefore, support stable gradient propagation in the input layer. Their role is numerical rather than physical, and they do not introduce additional information beyond what is already contained in the dataset. In contrast, the measured current (kA) is not redundant with respect to the current setting (%), as it reflects real process variability such as electrode wear, voltage fluctuations, and changes in contact resistance.

#### 2.6.2. ANN Model Architecture and Hyperparameters

**Figure 4:**
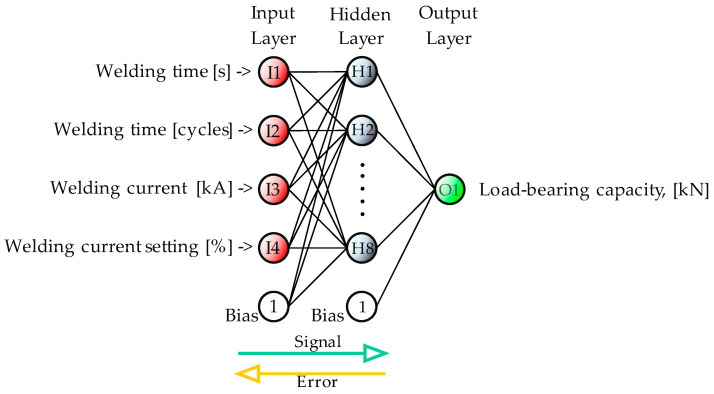
Topology of the neural network used.

The architecture and hyperparameter configuration of the ANN models were selected to provide a compromise between computational complexity and the accuracy of joint load-bearing capacity prediction. A multi-layer architecture with a 4-8-1 topology was employed, which allowed for methodological consistency while simultaneously accounting for the specific characteristics of different joint types. The input layer consists of 4 neurons, and one hidden layer contains 8 neurons, defining the network’s ability to extract nonlinear features from input data. The output layer consists of a single neuron generating a joint load-bearing capacity prediction expressed in kN. Bias neurons were introduced into both the input and hidden layers to support the stabilization of the learning process. The architecture diagram is presented in Figure 4. Various activation functions were used to transform the signals: a linear input, the hyperbolic tangent in the hidden layer, and a logistic output.

The optimization algorithm uses stochastic gradient descent (SGD), which updates the network weights based on calculated error gradients. The learning rate was set to 0.001, which ensures stable weight updates but may increase the model convergence time. Initial weights were randomly assigned from a uniform distribution in the interval [0, 1]. Mean square error (MSE) was used as a measure of the error between the prediction and the actual value. Batch Size was set to 1, meaning that the weights are updated after each individual training example. The training process was scheduled for a maximum of 1000 epochs across the entire dataset. An early stopping mechanism was used to prevent the model from overfitting the training data. The key hyperparameters are listed in Table 7.

The ANN architectures listed in Table 8 introduce a systematic comparative framework between models using two versus four input parameters. The introduction of variants with independent variables (Group A in Table 8) creates a set of models labelled A1, A2, and A3, each containing only two neurons in the input layer. In this configuration, the network uses exclusively the welding time (in cycles) and the current setting (in %). This design reduces the input space to strictly independent variables, enabling an objective assessment of whether the additional parameters provide meaningful information or merely constitute statistical ballast. Models in Group B (B1, B2, B3) retain all four input parameters. The baseline model used for comparison is the B2 network with a 4-8-1 topology.

Including all four parameters enables the network to capture fluctuations arising from factors such as electrode wear or voltage instability, thereby increasing the model’s robustness to process variability.

The developed ANN models provide a predictive framework capable of estimating the load-bearing capacity of resistance spot welded joints based on process parameters. Building upon these predictive capabilities, the next step of the study involved using the ANN output as the basis for optimizing welding parameters. Therefore, Section 2.7 intro-duces a genetic algorithm (GA) designed to identify parameter combinations that ensure high joint quality while reducing welding current and welding time within the tested range.

Due to the limited size of the experimental dataset, broad hyperparameter searches such as Random Search were found to produce unstable rankings of candidate architectures. Therefore, model selection was based on a controlled comparison of a small family of shallow feed-forward networks with comparable capacity. The 4-8-1 topology was selected because it consistently demonstrated the lowest variance across folds, smooth convergence behaviour, and robustness to weight initialization for all three joint configurations.

### 2.7. Optimization of RSW Process Parameters Using GA

To solve the decision problem, a genetic algorithm (GA) was implemented, which is a heuristic method for searching the state space, inspired by the mechanisms of natural evolution. The choice of GA was dictated by the nonlinear nature of the ANN model’s response function and the need to simultaneously consider multiple optimization criteria. Developing stable ANN-based predictive models provides the foundation for advanced optimization of technological parameters. In RSW processes, a key engineering challenge is not only ensuring the required mechanical strength of the joint, but also reducing welding current levels and shortening the production cycle time, factors that are particularly important from the manufacturer’s perspective due to their impact on production costs. This chapter presents the process of finding optimal settings for dissimilar joints (Grade 1/Grade 5), which, due to their thermophysical asymmetry, are characterized by the most complex dynamics of weld nugget formation.

#### 2.7.1. Genetic Algorithm Configuration

The algorithm operates on a population of individuals representing potential sets of parameters (current intensity and welding time). Through the successive application of selection, crossover, and mutation operators, the system strives to identify the solution that most effectively minimizes the objective function while maintaining the imposed technical constraints. The GA’s operating parameters were selected to enable efficient search of the solution space while avoiding local minima (high elitism and mutation rates). The GA specification is presented in Table 9.

#### 2.7.2. Formulation of the Optimization Problem

A key element of the process was defining a multi-criteria fitting function. The primary goal of the optimization was to find parameters that would guarantee high-quality joints (so-called “strong” joints), which under experimental conditions was defined as a load-bearing capacity exceeding the 3 kN threshold (F > 3 kN). At the same time, the objective function favored solutions characterized by:

- Minimizing current intensity. The lowest possible current intensity (I [kA] → min) at 50 cycles per second (t = Δp/50). Lower current settings reduce the thermal load on the electrodes and are generally associated with more stable operating conditions.
- Minimizing welding time. A shorter welding time results in a proportionally shorter process cycle within the tested parameter limits.

The genome structure in the applied genetic algorithm was limited to two decision genes: the welding current setting (Welding current setting [%]) and the welding time (Welding time [cycles]). This choice reflects the operating characteristics of the welding machine, as these are the only independent variables directly controlled by the operator. The genome structure is presented in Table 10.

For population generation, the ANN model variant B2 for G1/G5 joints was used. Although the ANN requires four input parameters, only two are independent in the optimization process, while the remaining two are treated as dependent:

- Measured current intensity [kA] is defined as a function of the current setting (I_measured_ = f(I_setting_)) rather than as an independent random variable. This prevents the GA from generating solutions that are mathematically valid but physically unattainable for the given welding machine.
- Welding time [s] is automatically converted from Welding time [cycles], ensuring temporal consistency of the model.

**Figure 5:**
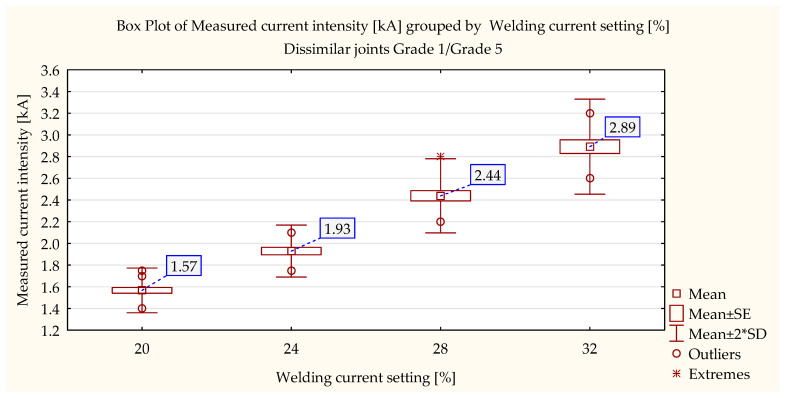
Experimental current characteristics of the welding machine.

Figure 5 presents the experimental current characteristic of the welding machine, illustrating the relationship between the operational setting (Welding current setting) and the measured current intensity (Measured current intensity) for the G1/G5 joint.

The observed strong linear correlation confirms the stability of the power supply system. The resulting transfer function was implemented in the hybrid GA–ANN structure. This reduced the genome dimensionality while preserving full physical consistency of the model, eliminating the risk of the genetic algorithm generating operating points that fall outside the current characteristics of the device.

The adopted search space boundaries were determined based on the technical capabilities of the welding device and the physicochemical conditions of the process, preventing the occurrence of undesirable phenomena such as excessive material spatter or overheating of the heat-affected zone (HAZ).

Constraints:

- Load-bearing capacity: F > 3 [kN],
- Measured current intensity [kA]: I_measured_ = f(I_setting_),
- Fitting function: f(t) = max(1/Δp + 1/I).

## 3. Experimental Results

### 3.1. Tensile-Shear Behaviour of Grade 1/Grade 1 Joints

**Figure 6:**
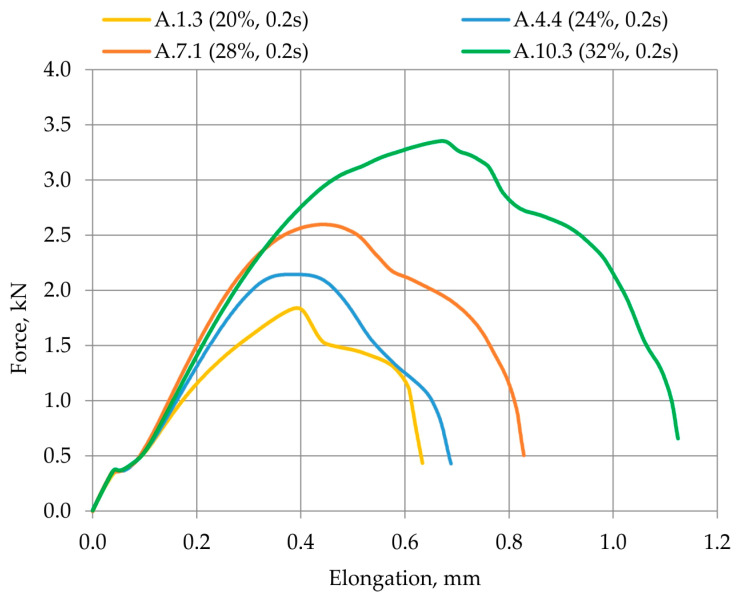
Force–elongation curves for Grade 1/Grade 1 joints.

Representative force–elongation curves for Grade 1/Grade 1 joints welded at a constant welding time of 0.2 s and varying current settings are presented in Figure 6.

An increase in welding current from 20% to 24% and 28% resulted in a gradual rise in the maximum load, occurring at an elongation of approximately 0.4 mm. At the highest current setting (32%), the maximum force further increased and was reached at a larger elongation of 0.7 mm.

**Figure 7:**
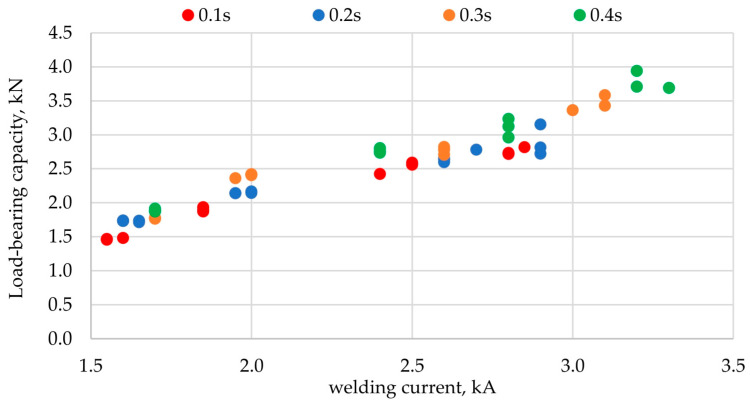
Load-bearing capacity of Grade 1/Grade 1 joints in the static tensile-shear test as a function of welding time and welding current.

**Figure 8:**
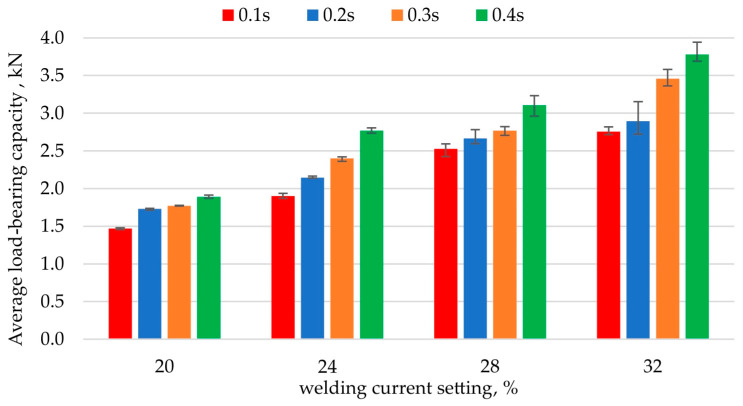
Average load-bearing capacity of Grade 1/Grade 1 joints for the welding parameter sets used in the study.

Comprehensive load-bearing capacity results for all parameter combinations are presented in Figure 7 and Figure 8.

Both higher welding current and longer welding time contributed to an increase in joint load-bearing capacity. The lowest average load-bearing capacity (1.47 kN) was obtained for a 20% current setting and welding time of 0.1 s. Extending the welding time at constant current increased the average load-bearing capacity by 18%, 20%, and 28% for welding times of 0.2, 0.3, and 0.4 s, respectively. The highest strength (3.78 kN) was achieved for 32% current setting and 0.4 s welding time.

**Figure 9:**
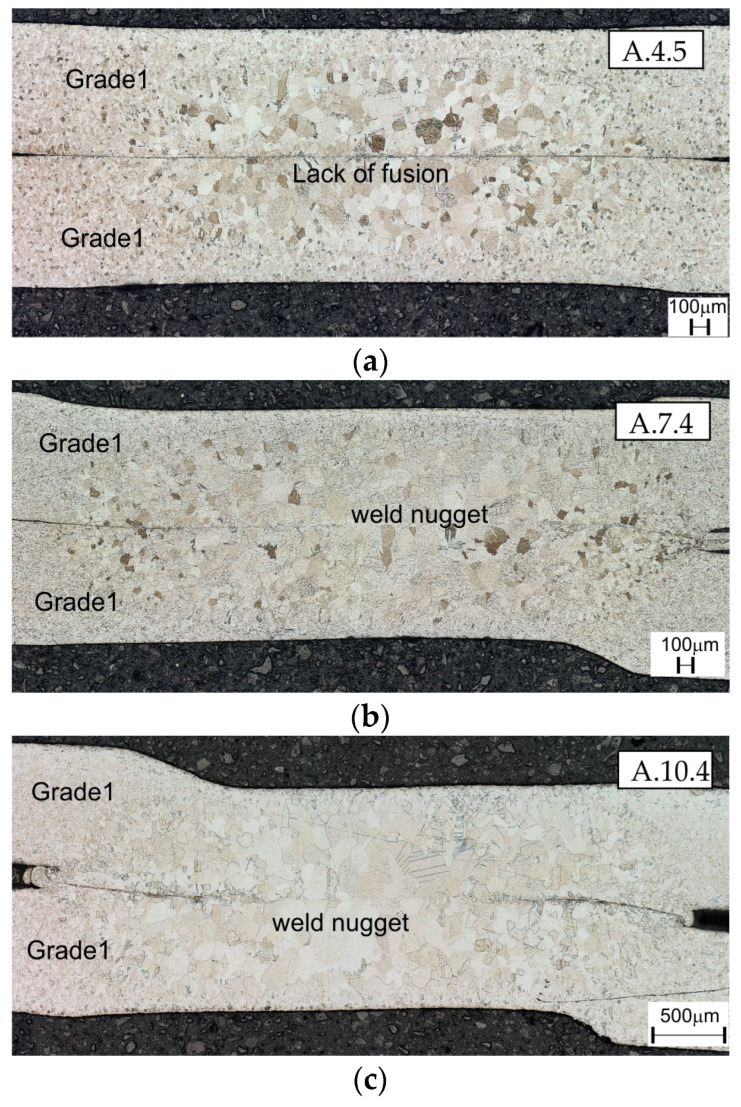
Microstructure of Grade 1/Grade 1 RSW joints: (**a**) welding current setting 24%, welding time 0.2 s; (**b**) welding current setting 28%, welding time 0.2 s; (**c**) welding current setting 32%, welding time 0.2 s.

Microstructures of selected Grade 1/Grade 1 joints are shown in Figure 9.

Insufficient welding parameters (low current or short time) did not provide enough heat input to form a weld nugget, resulting only in adhesion between the sheets (Figure 9a). Nugget formation was observed for higher current settings (28–32%) and adequate welding time (Figure 9b,c). Increasing the current and welding time widened the nugget and improved joint strength, which is consistent with tensile-shear test results.

### 3.2. Tensile-Shear Behaviour of Grade 5/Grade 5 Joints

**Figure 10:**
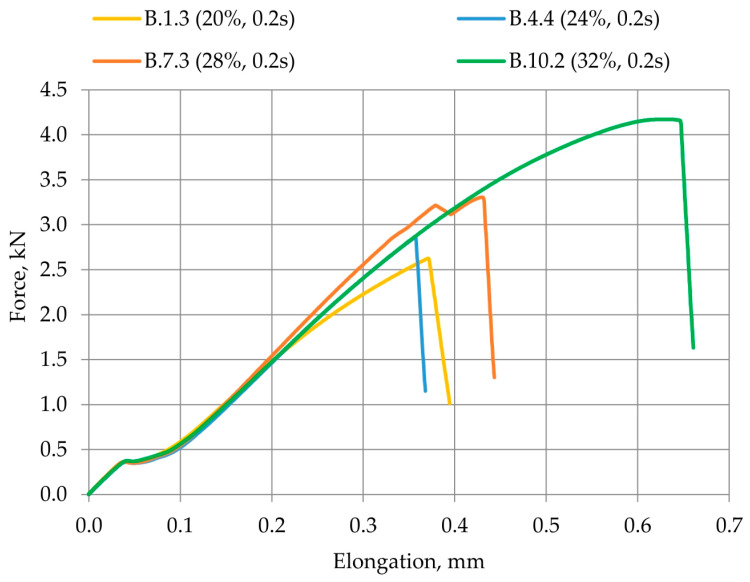
Force–elongation curves for Grade 5/Grade 5 joints.

Force–elongation curves for selected Grade 5/Grade 5 joints welded at 0.2 s are presented in Figure 10.

The lowest strength was recorded for the 20% and 24% current settings, where the maximum load did not exceed 3 kN and occurred at elongations below 0.4 mm. Increasing the current to 32% raised the maximum load above 4 kN.

**Figure 11:**
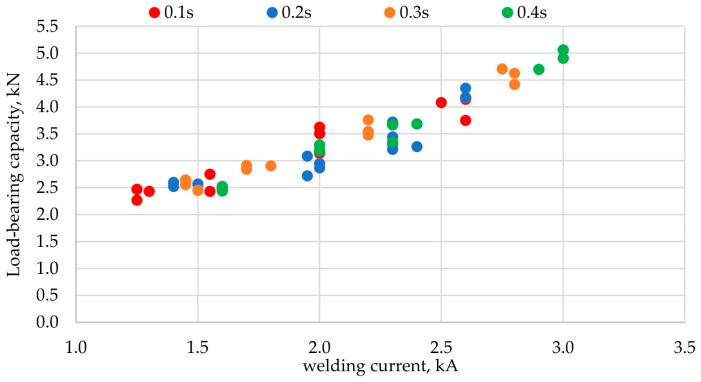
Load-bearing capacity of Grade 5/Grade 5 joints in the static tensile-shear test as a function of welding time and welding current.

**Figure 12:**
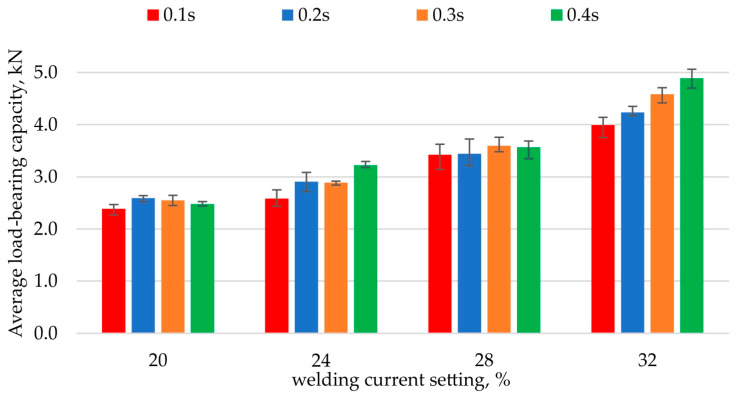
Average load-bearing capacity carried by Grade 5/Grade 5 joints for the individual welding current settings and welding times.

Figure 11 and Figure 12 summarize the results for all parameter sets.

The lowest load-bearing capacity (2.26 kN) was obtained for the joint welded at 1.25 kA and 0.1 s. Both higher current and longer welding time improved joint strength, with the highest value (5.06 kN) recorded for 3 kA and 0.4 s. The average load-bearing capacity for the strongest parameter set (32%, 0.4 s) was 4.88 kN.

**Figure 13:**
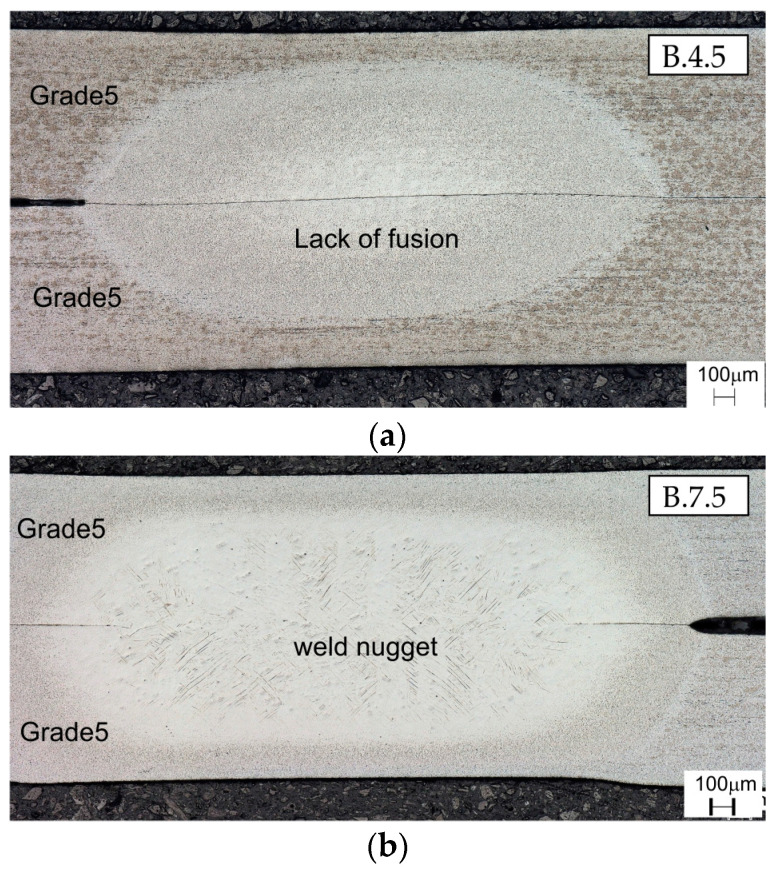

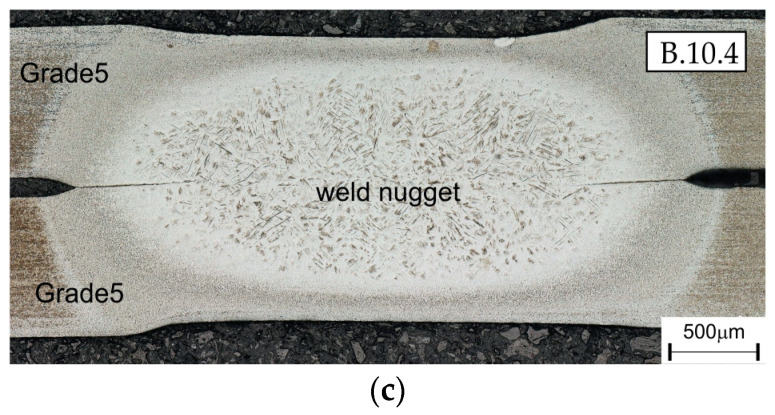
Microstructure of Grade 5/Grade 5 RSW joints: (**a**) welding current setting 24%, welding time 0.2 s; (**b**) welding current setting 28%, welding time 0.2 s; (**c**) welding current setting 32%, welding time 0.2 s.

Microstructures of selected Grade 5/Grade 5 joints are shown in Figure 13.

Low current and short welding time resulted in insufficient melting and the absence of nugget formation (Figure 13a). Proper nugget formation was observed for current settings ≥ 24% and welding times ≥ 0.2 s (Figure 13b,c). Increasing the heat input enlarged the nugget and improved the mechanical performance of the joints.

### 3.3. Tensile-Shear Behaviour of Grade 1/Grade 5 Joints

**Figure 14:**
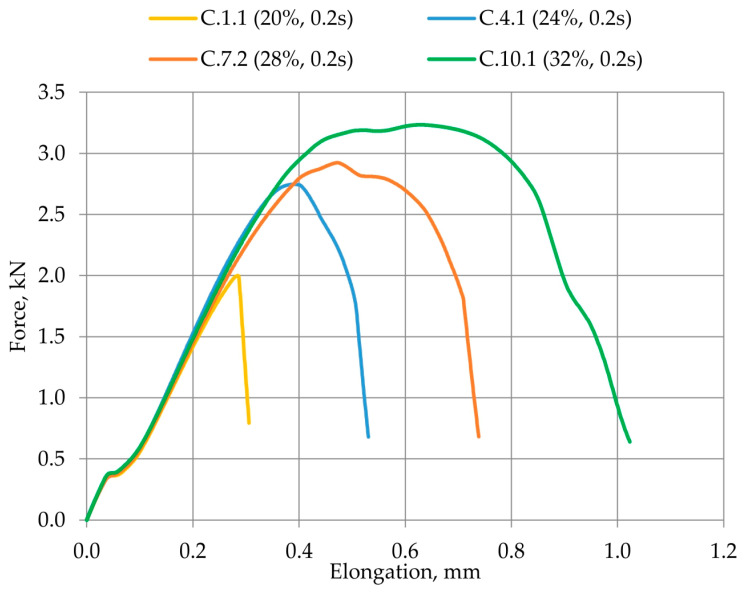
Force–elongation curves for Grade 1/Grade 5 joints.

Representative force–elongation curves for dissimilar Grade 1/Grade 5 joints are shown in Figure 14.

The joint welded at 20% current and 0.1 s failed at 1.95 kN and only 0.28 mm elongation. Increasing the current to 32% raised the maximum load to 3.2 kN and the elongation to 0.67 mm.

**Figure 15:**
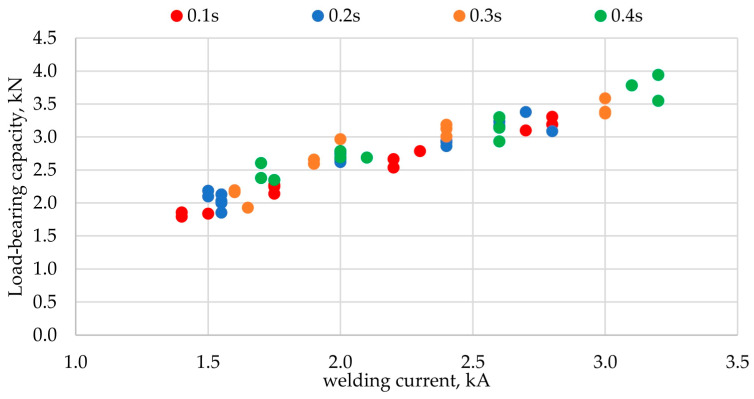
Load-bearing capacity by Grade 1/Grade 5 joints in the static tensile-shear test as a function of welding time and welding current.

**Figure 16:**
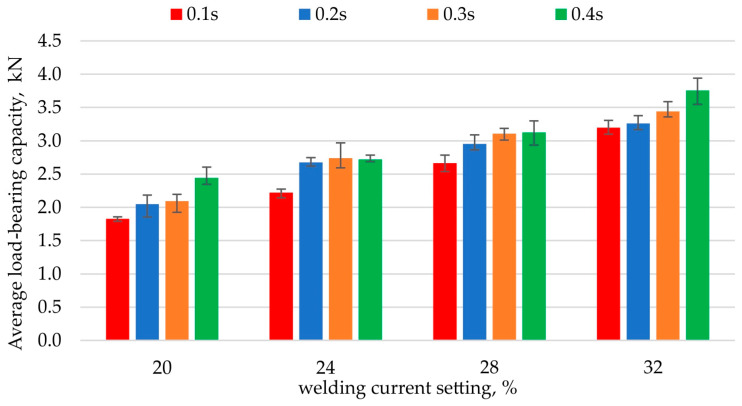
Average load-bearing capacity of Grade 1/Grade 5 joints for the individual welding current settings and welding times.

Figure 15 and Figure 16 present the complete dataset.

For a welding time of 0.1 s, the average load-bearing capacity increased from 1.82 kN (20%) to 3.19 kN (32%). Extending the welding time to 0.4 s increased the average load-bearing capacity by 33% (20% current), 22% (24% current), and 17% (28% and 32% current).

**Figure 17:**
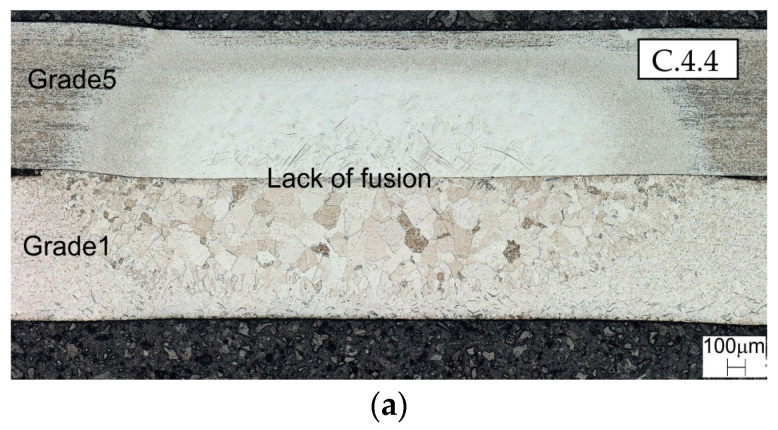

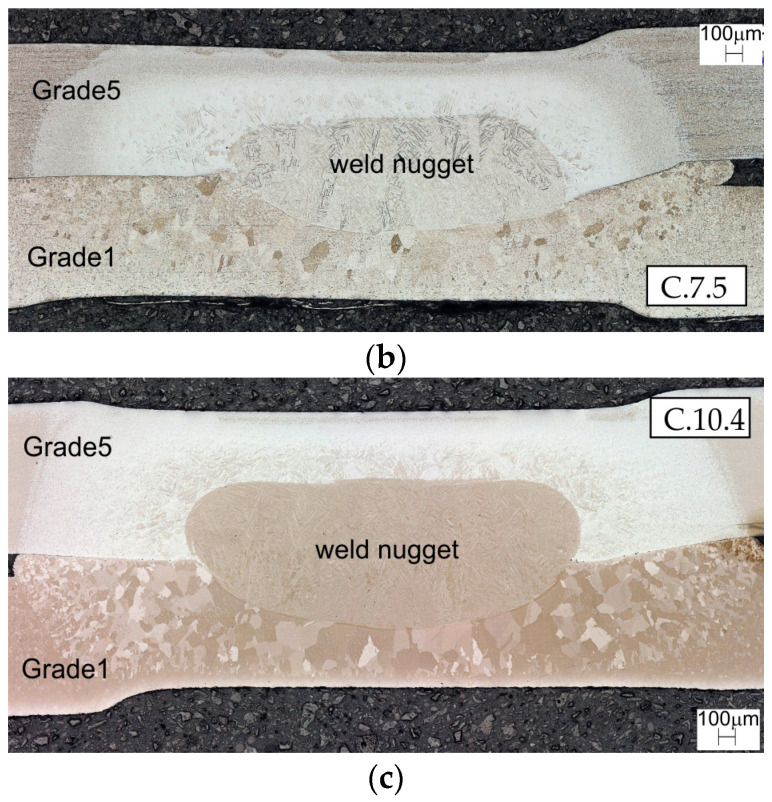
Microstructure of Grade 1/Grade 5 RSW joints: (**a**) welding current setting 24%, welding time 0.2 s; (**b**) welding current setting 28%, welding time 0.2 s; (**c**) welding current setting 32%, welding time 0.2 s.

Microstructures of selected dissimilar joints are shown in Figure 17.

For the current settings of 20% and 24%, no nugget was formed regardless of welding time (Figure 17a). Nugget formation began at 28% current and 0.1 s, and further increases in current and time enlarged the nugget. In dissimilar joints, the nugget was shifted toward the Grade 5 side, as visible in the micrographs (Figure 17c). This observation is qualitative and was not quantified, as such a measurement would not contribute meaningfully to the objectives of this study.

## 4. Model Learning and Validation Results

**Figure 18:**
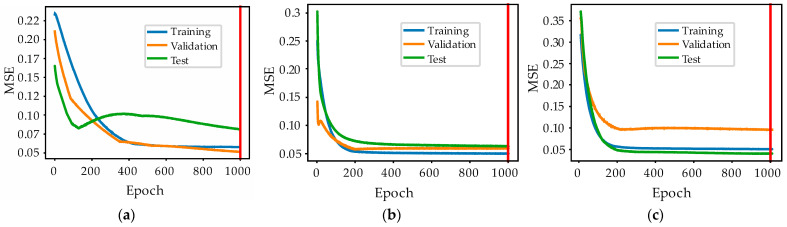
Mean square error (MSE) vs. number of epochs during training, validation, and testing of the models for individual joint types: (**a**) Grade 1/Grade 1; (**b**) Grade 1/Grade 5; (**c**) Grade 5/Grade 5.

The effectiveness of the learning process was monitored using the mean square error (MSE). Figure 18 shows the learning dynamics of the three neural network models by illustrating changes in MSE as a function of the number of epochs. These graphs are crucial for assessing the algorithm convergence and the quality of model fit to the data for each joint type. All three models (Figure 18a—Grade 1/Grade 1, Figure 18b—Grade 1/Grade 5, Figure 18c—Grade 5/Grade 5) demonstrate good learning characteristics. In the initial phase (the first 100–200 epochs), the MSE decreases rapidly, indicating effective weight optimization and fast network adaptation to the patterns contained in the training sets.

As shown in the graphs, after the initial sharp decline, the error curves flatten and reach asymptotic stability. This indicates that the models have achieved an optimal level of learning for the given architecture. A key aspect is the behaviour of the validation curve relative to the training curve. The graphs show that both curves follow a similar trajectory. The absence of a significant increase in validation error accompanied by a decrease in training error confirms that the models did not experience overfitting.

Although the curve shapes are similar, the models differ in their final error values. The Grade 1/Grade 5 model (Figure 18b) demonstrates a very stable training process and the lowest test error (0.065), making it the most reliable in the context of subsequent optimization using a genetic algorithm. The Grade 5/Grade 5 model (Figure 18c), despite stable training, exhibits the highest validation error (0.099), which may result from greater heterogeneity in the experimental data for this material grade. These graphs provide mathematical evidence that the networks were trained reliably. The low MSE values achieved during training (on the order of 0.05–0.09) are the basis for the high coefficient of determination, R^2^, presented in the subsequent figures. The final error values after 1000 epochs are summarized in Table 11.

**Figure 19:**
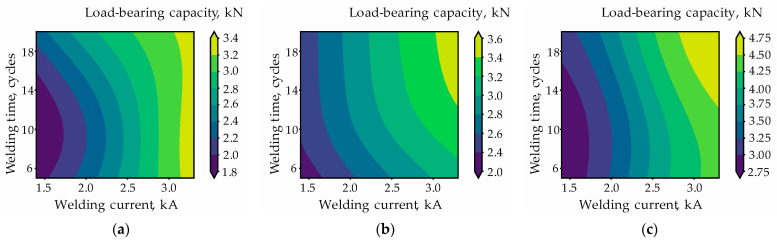
Prediction maps of joint load-bearing capacity, kN; (**a**) Grade 1/Grade 1; (**b**) Grade 1/Grade 5; (**c**) Grade 5/Grade 5.

Figure 19 presents a graphical interpretation of the neural network models in the form of three-dimensional prediction maps (response surfaces). These maps illustrate how simultaneous changes in current intensity and welding time affect the predicted joint load-bearing capacity. They make it possible to identify nonlinear relationships that are not captured by simple descriptive statistics.

The Grade 1/Grade 1 variant (Figure 19a) demonstrates a relatively smooth increase in load-bearing capacity with increasing parameter values. A stable plateau is visible at medium current levels, suggesting a wide technological window for this joint configuration. The Grade 1/Grade 5 configuration (Figure 19b) is characterized by the greatest dynamics of change. Distinct regions of high load-bearing capacity appear, indicating high sensitivity of this joint to precise parameter selection. This model was, therefore, selected for further optimization using a genetic algorithm.

The Grade 5/Grade 5 homogeneous lap joint configuration (Figure 19c) is the most irregular, which correlates with the higher validation error reported in Table 11. This suggests that welding Grade 5/Grade 5 titanium is a process with higher variability in results.

A visual analysis of all three maps shows that the horizontal axis, representing current intensity [kA], generally has a stronger influence on the surface slope than the time axis. This means that even a small change in current intensity causes a more rapid increase (or decrease) in joint strength than a change in welding time. The highest load-bearing capacity values (marked in yellow/white on the maps) occur in the upper-right corner of the graphs, corresponding to combinations of high current and long time.

However, the maps also make it possible to identify “safe” points where acceptable load-bearing capacity is achieved at lower parameter values (green/light-green areas). Prediction maps can function as technological nomograms: they allow the engineer to visually assess process risk—areas with a steep slope (rapid color transitions) represent unstable zones where even a slight setting error may lead to a drastic reduction in joint quality. They also enable the determination of load-bearing capacity isolines, i.e., different combinations of current and time that yield the same strength, which allows for the optimization of energy consumption or cycle time.

### 4.1. Correlation Scatter Analysis and Statistical Verification

**Figure 20:**
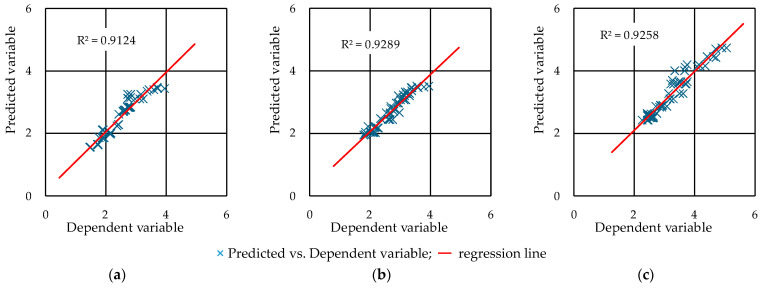
Correlation scatterplots of the joint load-bearing capacity: (**a**) Grade 1/Grade 1; (**b**) Grade 1/Grade 5; (**c**) Grade 5/Grade 5.

To finally assess the predictive capabilities of the developed models, experimentally obtained load-bearing capacity values were compared with the results generated by the neural networks. These relationships are presented in the correlation scatterplots (Figure 20), which illustrate the degree of model fit to the actual data. Statistical analysis, summarized in Table 12, confirms the high precision of the models. For all analyzed joints, the R^2^ value exceeded 0.91, reaching a maximum of 0.9289 for the Grade 1/Grade 5 joint.

The mean absolute error (MAE) did not exceed 0.15 kN, while the mean relative error (MRE) remained low, ranging from 4% to 6% depending on the joint configuration. The highest deviation of the maximum absolute error (E_max_) was recorded for the Grade 5/Grade 5 joints (0.66 kN), which translates to a 20% maximum relative error (R_max_). The lowest value (0.42 kN) was recorded for the Grade 1/Grade 5 joints. The largest minimum absolute error (E_min_) did not exceed 0.01 kN, which translates to 0% of the minimum relative error (RE_min_). The correlation graphs (Figure 20a–c) show that most measurement points are concentrated in the immediate vicinity of the regression line, demonstrating that the ANN models correctly captured the non-linear relationships between the welding parameters and the joint mechanical strength.

Table 12 provides a statistical summary of the prediction quality of the developed ANN models. This information is crucial for assessing the network’s reliability, as it compares the computational errors with actual experimental results. All models achieved very high values of the coefficient of determination R^2^, exceeding 0.91. The highest fitting precision (0.9289) was obtained for the Grade 1/Grade 5 mixed-joint model, confirming the validity of selecting this network for further optimization using a genetic algorithm.

The mean relative error values range from 4% to 6%. This means that the average deviation between the predicted and actual load-bearing capacity is only about 5%, which is considered highly accurate in welding process studies. The maximum absolute error reflects the largest single deviation within the entire data set. The smallest error (0.42 kN) was recorded for the Grade 1/Grade 5 joints, demonstrating the high repeatability of this model’s predictions. The largest error (0.66 kN) occurred in the Grade 5/Grade 5 model, which correlates with the higher validation error in Table 11.

### 4.2. Results of Two-Level Factorial Design (2^k^)

The two-level 2(2-0) factorial design successfully identified linear relationships and interactions affecting the load-bearing capacity of titanium joints. All models obtained using this design demonstrate good fit (R^2^ ranging from 0.87 to 0.93) and relatively low Mean Absolute Percentage Error (MAPE) (5.3–6.5%). These models are considered valid because the *p*-value for the Lack of Fit error for each model exceeds 0.05, indicating no significant fit error. Analysis of the F statistics and Pareto charts for all configurations confirms that welding current intensity is the factor with the greatest influence on the joint’s load capacity. It always has the highest F statistic value (e.g., F = 286.18 for G1/G1), making it a key parameter for precise control in the production process.

As shown in the ANOVA analysis presented in Table 13, G1/G1 welded joints exhibit the highest coefficient of determination (R^2^ = 0.92558) for the two-level factorial design analyses. Both (1) Welding current setting % and (2) Welding time cycles have a critical impact on the results (*p* = 0.000). Interaction between current and time (1 by 2), checks whether the influence of one parameter changes depending on the value of the other (if *p* < 0.05, the interaction is significant). A statistically significant (1 by 2) interaction was observed (*p* = 0.003656), indicating that the effect of current intensity on the load capacity of the G1/G1 joint changes with welding time. Degrees of freedom (df) is the number of independent pieces of information used to calculate a given statistic, in this case, df = 29. The Snedecor F-test statistic (F) is used to assess the significance of a factor’s influence (the higher the F value, the greater the parameter’s influence on the load capacity). In the ANOVA model, the Mean Square (MS) and MS Pure Error are fundamental components used to evaluate the significance of a model and the presence of Lack of Fit. In the ANOVA model, the variable (Var.) represents the load-bearing capacity, kN, i.e., the test result. Sum of squares (SS) represents the sum of squared deviations, i.e., a measure of the total variability attributed to a given factor, while total sum of squares (Total SS) represents the total variability observed across all trials.

**Figure 21:**
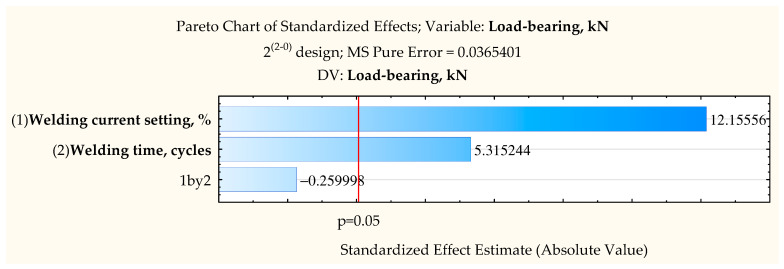
Pareto chart of standardized effects for G1/G5 joints using factorial design.

For the G1/G5 welded joint, the model achieved a fit of R^2^ = 0.87264. Figure 21 shows that, as in the case of pure titanium, both main parameters are significant, but the interaction between them is insignificant (*p* = 0.796994). This suggests that in dissimilar joints, these parameters act more independently of each other within the studied range.

**Figure 22:**
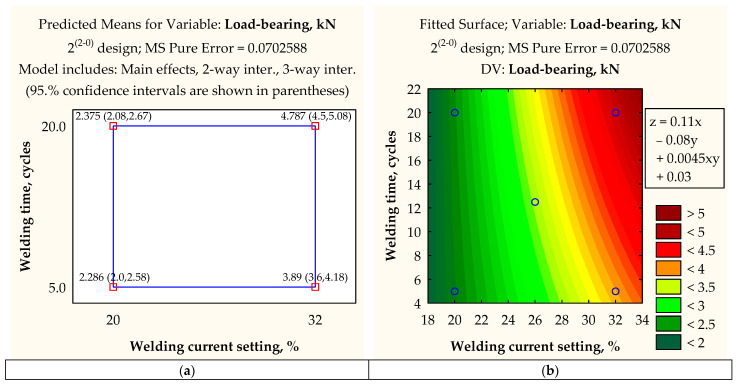
Prediction of the load-bearing capacity, kN of G5/G5 joints as a function of welding parameters using factorial design: (**a**) point average analysis; (**b**) contour map of the response surface.

For the G5/G5 joint, the coefficient of determination is R^2^ = 0.87159. Current remains the dominant factor (F = 172.15), and the effect of welding time, although still significant (*p* = 0.003530), is much weaker than for Grade 1 joints. The interaction of parameters is statistically significant (*p* = 0.014161). Figure 22 presents a graphical interpretation of the statistical model for G5/G5 joints, developed based on a two-level factorial design. It consists of two complementary parts that illustrate how process parameters affect the load capacity (expressed in kN). Figure 22a presents the predicted load capacity values at key points in the experimental design. This allows for a quick assessment of the differences in joint strength at low and high parameter settings. Figure 22b is a visualization of the regression plane, which allows for the predicted load capacity for any combination of parameters in the studied area. Importantly, this model is characterized by a lack of significant fit error (Lack of Fit *p* = 0.100055), confirming that the graphical representation of the relationships in Figure 22 is statistically accurate and reliable.

Table 14 summarizes the statistical indicators assessing the quality and accuracy of the model fit obtained using a two-level factorial design for three types of titanium joints for a sample size of n = 30. The highest value was observed for G1/G1 joints (R^2^ = 0.92558), meaning that the model explains over 92% of the variability in the load capacity results. For the G1/G5 and G5/G5 joints, these values fluctuate around 87%, which still indicates a very good fit of the model to the experimental data.

The Pearson correlation coefficient (r) for all models exceeds 0.93 (reaching 0.96 for G1/G1), confirming a very strong linear relationship between the process parameters (current and time) and the joint strength. The MAPE ranges from 5.3% (for the G1/G1 and G1/G5 joints) to 6.5% (for G5/G5). In engineering practice, an error at this level is considered evidence of high accuracy of the predictive model. RMSE (Root Mean Square Error) values range from 0.1582 kN to 0.2471 kN. They indicate the average deviation of the model’s predicted load capacity from the actual results obtained in the destructive tests.

### 4.3. Results of Central Composite Design (CCD) and Response Surface Method (RSM) Analysis

The ANOVA for the G1/G1 welded joint shown in Table 15 demonstrates the highest coefficient of determination, R^2^ = 0.9682 (${\bar{R}}^{2}$ = 0.96481), meaning that the model explains nearly 97% of the variability in the results. The linear parameters of current and time have a key impact on load capacity (*p* = 0.000), but the interaction between them (1L by 2L) and the quadratic effect of current (*p* = 0.012471) are also significant.

**Figure 23:**
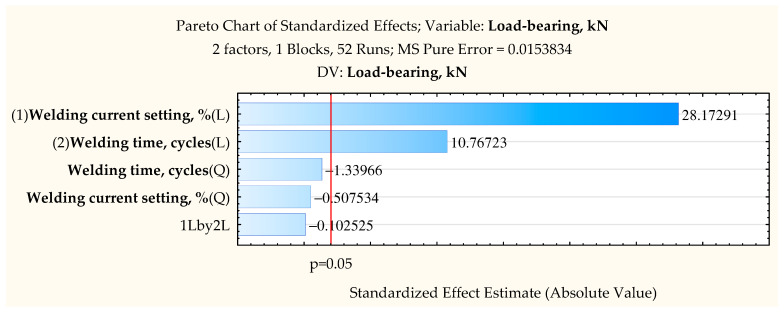
Pareto chart of standardized effects for G1/G5 joints using CCD.

The model for the G1/G5 welded joint demonstrates a coefficient of determination of R^2^ = 0.94124. In this configuration (Figure 23), only the linear effects of current and time dominate. The quadratic effects and the interaction did not reach statistical significance (*p* > 0.05), suggesting a more linear nature of strength increase in the studied technological window for dissimilar joints.

**Figure 24:**
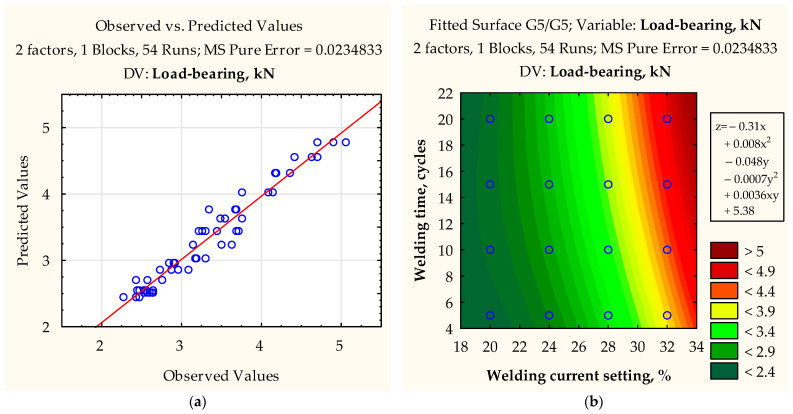
Prediction of the load-bearing capacity, kN of G5/G5 joints as a function of welding parameters using CCD: (**a**) predicted vs. observed values; (**b**) contour map of the response surface.

Figure 24a shows a plot of predicted versus observed values for the load-bearing capacity of joints made of G5/G5 alloy. This plot is used to graphically assess the quality of fit of the model developed using the CCD method. The clustering of measurement points is close to the line of perfect fit. For the G5/G5 welded joint, the model achieved R^2^ = 0.95167. Figure 24b shows the contour map of the response surface for homonymous joints made of Grade 5 titanium alloy. The figure and the associated ANOVA statistical analysis indicate that welding current (horizontal axis) has a stronger effect on the surface slope (i.e., the rapidity of the load capacity change) than welding time. The contour lines reflect a very strong quadratic effect of current (F = 39.945; *p* = 0.000) and a statistically significant interaction between current and time (*p* = 0.000178). This indicates that the effect of one parameter on joint quality varies depending on the value of the other parameter and is nonlinear in nature. The highest load capacity values, reaching up to 5.06 kN in the experimental tests, are visible in the upper right corner of the map, corresponding to the simultaneous use of high current and long welding time.

CCD models are characterized by high predictive precision, as confirmed by the statistical indicators assessing the quality and accuracy of CCD matrix matching, presented in Table 16. MAPE: This ranges from 3.5% (for G1/G1) to 4.0% (for G5/G5), which is considered accurate in welding process studies. RMSE: The load-bearing capacity prediction error ranges from 0.11 to 0.16 kN, allowing for accurate joint strength estimation.

However, it should be noted that CCD models, despite their high R^2^ > 0.94 and adjusted coefficient of determination ${\bar{R}}^{2}$ > 0.93, exhibit a statistically significant lack of fit error (Lack of Fit *p* < 0.05). This results from the very high repeatability of measurements at the center points. In such cases, the Lack of Fit test becomes overly sensitive, indicating a model misfit that is negligible from an engineering perspective and does not impact the predictive ability of the developed response surface. Moreover, this situation may suggest that, in the RSW process under study, additional complex phenomena may be present that are not fully captured by the quadratic model.

### 4.4. Comparative Analysis of Neural Network Models

The comparative analysis was performed on B2 baseline networks with a 4-8-1 topology. The results presented in Table 17 provide empirical evidence that verifies the validity of using duplicated input parameters in neural network (ANN) models. Table 17 allows for a direct comparison of the performance (Group A vs. Group B) and shows whether the additional parameters actually add value to the model. The numerical data indicate that the variants with 4 inputs (Group B) achieve higher accuracy than their counterparts from Group A. For G1/G5 joints, the B2 (4-8-1) model achieved R^2^ = 0.92885 and the lowest MAPE = 4.3%. In comparison, the A2 (2-8-1) model for the same joint type reached R^2^ = 0.91177 and MAPE = 4.7%, demonstrating improved precision when redundant parameters are included. For G1/G1 joints, the B3 (4-16-1) model achieved R^2^ = 0.92386 and MAPE = 5.4%, whereas the A3 (2-16-1) model showed lower agreement (R^2^ = 0.90913) and higher error (MAPE = 6.6%). The B2 baseline networks with the 4-8-1 topology used for comparison are bolded in Table 17.

The comparison between Group A (two inputs) and Group B (four inputs) confirms that the additional inputs do not merely increase fitting flexibility. Group B models exhibit lower variance across folds and more stable convergence, indicating improved numerical conditioning rather than overfitting to repeated points. The two representations of welding time contribute different numerical scales after normalization, which supports stable gradient propagation in small datasets. In contrast, the measured current (kA) is not redundant with respect to the current setting (%), as it captures real process variability and, therefore, provides additional physical information.

The selection of the final B2 architecture was guided by its reproducibility across all joint configurations. Among all tested topologies, the 4-8-1 model was the only one that consistently maintained low fold-to-fold variance and stable convergence behaviour, which made it the most reliable choice for the predictive framework.

The higher statistical parameters of Group B indicate that the additional inputs do not artificially improve the fit but provide the network with meaningful information about physical fluctuations in the process. Although the time expressed in cycles and seconds represents the same quantity, Table 17 shows that supplying both inputs stabilizes the learning process and enables higher precision (lower RMSE and MAPE) in processes where time control is critical. The results demonstrate that the simplified model (Group A) is correct, but the extended model (Group B) is more effective at extracting nonlinear features from the data, which translates into lower prediction errors (MAE, MAPE) and stronger correlation.

### 4.5. Comparative Performance Analysis of K-Fold Cross-Validation for B2 Models

To evaluate the robustness and generalization capability of the proposed B2 ANNs, a comprehensive k-fold cross-validation (k = 10) was conducted across three distinct technological datasets: B2(G1/G1), B2(G1/G5), and B2(G5/G5). This procedure ensured that the predictive accuracy was not biased by the specific partitioning of training and testing data.

The load-bearing ranges reported in Table 18 correspond to the individual datasets for each joint configuration (G1/G1, G1/G5, G5/G5). These ranges differ because each material pair exhibits a distinct mechanical response. The overall experimental range across all tested joints (1.45–3.06 kN) is, therefore, wider than the per-dataset ranges used in the cross-validation analysis.

The performance metrics, including MSE, Root Mean Squared Error (RMSE), and the standard deviation of the mean squared error (SD_MSE_), are summarized in Table 18. The term “estimated” R^2^ is also used. This means that the coefficient of determination was not calculated for the entire dataset at once, but represents an average value obtained from the cross-validation procedure. The estimated R^2^ is the mean of 10 results, because in each iteration, the model was tested on data it had not previously seen.

The results indicate that the ANN model achieves high fidelity across all tested scenarios. The highest absolute precision was observed for the B2(G1/G1) dataset, with an RMSE of only 0.0752 kN. Even for the highest load-bearing configuration B2(G5/G5), the error remained low at 0.1102 kN, reflecting a high signal-to-noise ratio in the ANN predictions. The mean MSE for B2(G1/G5) of 0.055 at load-bearing capacities ranging from 1.8 to 2.6 kN indicates that the model performs well when predicting data it had not encountered during training. The very low standard deviation of the MSE across all folds (SD_{MSE} ranging from 0.0008 to 0.0051) confirms the numerical stability of the network architecture. This demonstrates that the neural network responds reproducibly and is resistant to overfitting. The model is insensitive to specific selection of training data and maintains consistent performance regardless of the subset used.

In the case of the B2(G1/G1) data set, a significant improvement in the statistical parameters of the model was observed compared to the B2(G1/G5) dataset. The mean MSE was reduced to 0.00565 (kN^2^), while maintaining a very low dispersion between individual validation iterations (SD = 0.00078). This indicates the highly deterministic nature of the processes represented in the B2(G1/G1) dataset.

The RMSE was determined to provide a physical interpretation of the prediction error. For the B2(G1/G5) dataset, the mean model error is 0.235 kN, which, at maximum load capacities of 2.6 kN, represents a relative error of approximately 9%. For the B2(G1/G1) dataset, the model demonstrated nearly three times higher precision, achieving an RMSE of 0.075 kN.

The consistency of errors across varying capacity ranges (from 1.45 kN to over 3.0 kN) demonstrates that the ANN effectively captures the underlying physical relationship between the welding parameters (current intensity and time) and the resulting structural integrity. The cross-validation study confirms that the developed neural network can serve as a reliable surrogate model for real-time load-bearing capacity estimation.

In addition to the record-level k-fold procedure, a grouped cross-validation scheme was performed to eliminate the possibility that repeated measurements obtained under identical welding conditions could appear simultaneously in the training and validation subsets. In this approach, all specimens produced with the same combination of welding current and welding time were kept together within a single fold. This method provides a stricter assessment of the model’s ability to generalize between different technological settings. The grouped validation confirmed that the ANN does not rely on memorizing repeated points and that the learned relationship between process parameters and load-bearing capacity remains consistent across folds. A detailed quantitative analysis of the grouped validation results is presented in Section 4.6.

### 4.6. Grouped K-Fold Cross-Validation Strategy

To complement the record-level validation and to assess the model’s ability to generalize to previously untested welding conditions, a Grouped K-Fold cross-validation scheme (8 folds) was applied. In this approach, all specimens produced under the same combination of welding current setting and welding time were assigned to a single group (16 groups per dataset). This prevents data leakage between training and validation subsets and provides a stricter evaluation of the model’s predictive capability for unseen technological settings.

The grouped validation revealed a systematic increase in prediction error relative to the standard K-Fold procedure (Table 19). This effect was most pronounced for the G1/G1 configuration, where the RMSE increased from 0.075 kN (record-level CV) to 0.223 kN. The decrease in accuracy reflects the limited variance of the load-bearing capacity within this dataset and the sparsity of the experimental grid. When the model is required to extrapolate beyond the local neighbourhood of the training points, the prediction uncertainty naturally increases.

The negative R^2^ observed for the G1/G1 configuration is consistent with the statistical properties of this dataset. The load-bearing capacity values are highly clustered, resulting in a very small denominator in the R^2^ definition. Under such conditions, even minor deviations between predicted and measured values may cause the metric to fall below zero. This behaviour suggests that, for homogeneous Grade 1 joints, the model’s ability to generalize to entirely new parameter combinations is limited by the narrow dynamic range of the response variable.

In contrast, the mixed-grade configuration (G1/G5) exhibits a higher R^2^ (0.30) under grouped validation. The broader variability of mechanical response in dissimilar joints provides a more informative gradient for the model, enabling more reliable extrapolation to unseen welding conditions.

### 4.7. Optimal Set of Technological Parameters

The best result for the given optimization problem turned out to be the individual with the parameters presented in Table 20, which provides a key summary of the entire research process, presenting the optimal set of technological parameters determined by the genetic algorithm (GA) for the Grade 1/Grade 5 dissimilar joint. The algorithm identified a specific combination of variables that most effectively satisfied the assumptions of the fitness function. The welding current (2.89 kA) is the highest value within the tested range. The algorithm also selected the shortest available welding time (5 cycles, i.e., 0.1 s) within the search limits. This result is advantageous from a process-practical standpoint, as it corresponds to the shortest operating cycle available within the tested parameter range.

The obtained value is consistent with the adopted threshold criterion (F > 3 kN). The algorithm did not seek the highest possible strength, but the lowest current-time parameters that would guarantee the required quality.

**Figure 25:**
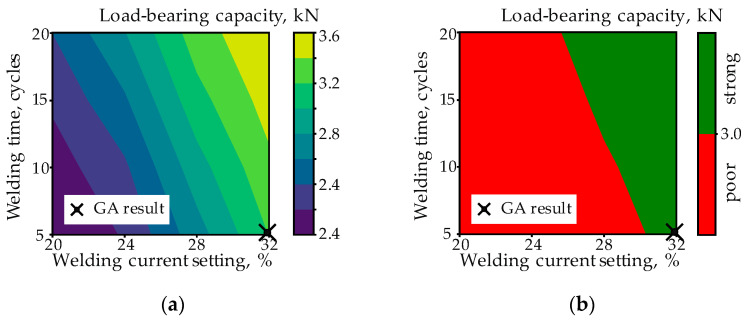
GA optimization results: (**a**) prediction map of joint load-bearing capacity; kN; (**b**) map of weak and strong joints.

Figure 25 presents a graphical interpretation of the results in the form of contour maps. The load-bearing capacity prediction map in Figure 25a illustrates the predicted joint strength as a function of welding current and time. The map colors correspond to the load-bearing capacity values ranging from 2.40 kN to 3.60 kN. The qualitative classification of the joints is shown in Figure 25b. The figure shows a binary division of the parameter space into low-strength joints, marked in red, and high-strength joints, marked in green. The division boundary is the load-bearing capacity criterion F > 3 kN assumed in the optimization problem.

In both graphs, the symbol “X” indicates the parameters of the best individual determined by the algorithm. The optimal solution corresponds to a current intensity of 2.89 kA and a minimum welding time of 5 pulses (0.1 s). This point is located close to the border of the “strong” region, which is confirmed by the obtained load-bearing capacity of 3.2 kN. This choice is consistent with the assumed fitness function, which aimed to minimize welding current and operation time while maintaining the required joint strength. The map also shows that although it is possible to obtain higher load capacities (yellow areas in Figure 25a), this would require an increase in the current intensity or an extension of the welding time, which would be inefficient from the point of view of process optimization.

The optimal parameter set identified by the GA represents a model-based optimum derived from the validated ANN predictor. Due to experimental constraints, this operating point was not verified in an additional welding trial and is, therefore, proposed as a recommended region for future confirmatory testing.

## 5. Conclusions

Based on the experimental investigations, numerical modeling, and optimization results presented in this study, the following conclusions can be drawn:

- Welding parameters strongly influence joint quality. Insufficient heat input, resulting from low current or short welding time, led to incomplete nugget formation and low load-bearing capacity. Increasing current and time consistently improved joint strength.
- Material grade determines the achievable strength level. Grade 5/Grade 5 joints exhibited the highest strength due to the superior mechanical properties of Grade 5 (Ti6Al4V), while dissimilar joints showed intermediate strength.
- Artificial neural networks accurately captured nonlinear process behavior. The developed 4-8-1 ANN models demonstrated high predictive capability, with determination coefficients above 0.91 and relative errors below 6% for all material configurations.
- Genetic algorithm optimization enabled the identification of efficient welding parameters. For the dissimilar Grade 1/Grade 5 joint, the required load capacity above 3 kN was achieved with a minimum welding time of 5 cycles (0.1 s) and a current of 2.89 kA, representing an optimal balance between strength and reduced welding current and time.
- The developed prediction and quality maps provide a practical engineering tool. They allow rapid assessment of the influence of current and time settings on joint strength without the need for destructive testing.
- Future work will focus on addressing the key aspects related to welding current stability and measurement accuracy, including the implementation of improved current monitoring and stabilization strategies. Moreover, future work will include an experimental verification of the GA-predicted optimum to confirm its practical applicability under industrial welding conditions.

## Figures and Tables

**Table 1:** Chemical composition of titanium Grade 1 [Inspection Certificate No. 3071/2022, Nippon Steel & Sumitomo Metal Corporation, Joetsu, Japan, 20 January 2022].

Element	O	H	Fe	Ti
Content (wt.%)	0.03	0.001	0.03	bal

**Table 2:** Chemical composition of titanium alloy Grade 5 [Inspection Certificate No. 2010023, 12 January 2010].

Element	Al	V	Fe	O	C	N	H	Ti
Content (wt.%)	5.5–6.75	3.5–4.5	≤0.40	≤0.20	≤0.08	≤0.05	≤0.015	bal

**Table 3:** Mechanical properties of analysed sheets.

Material	Offset Yield Point R_p0.2_, MPa	Tensile Strength R_m_, MPa	Elongation A_10_, %
Grade 1	240	302	41
Grade 5	970	1000	12

**Table 4:** Technical specifications of the TECNA 4640 welding machine.

Parameter	Value
Rated power (50%) [kVA]	16
Maximum power [kVA]	35
Secondary voltage [V]	3.4
Maximum short-circuit current [kA]	12.5
Supply voltage [V]	400
Supply frequency [Hz]	50
Maximum clamping force [N]	2400

**Table 5:** Welding process parameters.

Welding Current Setting [%]	Welding Time [Cycles] (1 Cycle = 20 ms)	Electrode Force [N]
20, 24, 28, 32	5, 10, 15, 20	2400

**Table 6:** Characteristics of data sets for ANN models.

Sample Type	Number of Data	Training Set	Test Set	Validation Set
Grade 1/Grade 1	53	78%	11%	11%
Grade 1/Grade 5	52	75%	13%	12%
Grade 5/Grade 5	54	78%	11%	11%

**Table 7:** ANN hyperparameters.

Hyperparameter	Value	Concise Description
Learning Rate (α)	0.001	Controls the step size during optimization. A higher value speeds up learning but may cause instability, while a lower value ensures stability but slows training.
Batch Size	1	The number of training examples used in one forward and backward pass. A larger batch improves stability but requires more memory.
Epochs	Max 1000	The number of times the entire dataset is passed through the network during training. More epochs can improve accuracy but may lead to overfitting.
Weight Initialization	Random Initialization	Assigned small random values from a uniform distribution in the interval [0, 1].
Activation Function	Input: Linear Hidden: Tanh Output: Logistic	Defines how neurons transform input signals.
Regularization	Early Stopping	Prevents overfitting by not training for too long.
Optimizer	SGD	Stochastic Gradient Descent, algorithm that updates weights based on gradients.
Number of Hidden Layers	1	Determines the depth of the network, affecting its ability to learn complex patterns.
Number of Neurons per Layer	Input: 4 Hidden: 8 Output: 1	Defines the capacity of each layer to extract features from input data.
Loss Function	MSE	Measures the error between predictions and actual values

**Table 8:** Summary of ANN topology parameters.

ID	Input Layer Neurons	Description of Input Neurons	Hidden Layer Neurons	Output Layer	Output Description	Total Neurons
A1	2	Welding time [cycles] Welding current setting [%]	4	1	Load-bearing capacity [kN]	7
A2			8			11
A3			16			19
B1	4	Welding time [s] Welding time [cycles] Welding current setting [kA] Welding current setting [%]	4			9
B2			8			13
B3			16			21

**Table 9:** GA hyperparameters.

Hyperparameter	Value/Range/Options	Description Comments
Population Size	40	Number of individuals in each generation.
Genome Size	2	Number of genes in an individual’s genome.
Encoding Scheme	Real-valued	Representation of the decision variables.
Number of Generations	20	Total iterations the GA will run through to evolve solutions. Total number of generations before stopping
Selection Method	Tournament	How individuals are chosen for reproduction.
Tournament Size	4	Number of individuals randomly chosen to compete for selection.
Crossover Type	One-point	Type of crossover used (e.g., one-point, two-point, uniform)
Crossover Rate	1	Probability of crossover occurring between parents.
Mutation Type	Swap	Type of mutation applied (e.g., bit flip, Swap, Gaussian noise)
Replacement Strategies	Elitism	The best individuals from the previous generation are preserved in the new population.
Elitism Rate	25%	The percentage of the best individuals in a population that is retained in the next generation without modification.
Termination Criteria	Fixed generations	Reaching the set number of generations

**Table 10:** GA genome information.

Parameter Name	Gene Name	Parameter Range	Parameter Value Step
Welding current setting	I_setting_	20–32	4
Welding time [cycles]	t_cycles_	5–20	5

**Table 11:** Results of the network training process.

ID	Lap Joint Configuration	Number of Epochs	Training MSE	Validation MSE	Test MSE
1	Grade 1/Grade 1	1000	0.06	0.051	0.079
2	Grade 1/Grade 5	1000	0.05	0.064	0.065
3	Grade 5/Grade 5	1000	0.051	0.099	0.047

**Table 12:** Statistical data from the obtained results of the artificial neural network.

ID	E_max_	E_min_	MAE	RE_max_	RE_min_	MRE	R^2^
1	0.52	0.01	0.15	19%	0%	6%	0.9124
2	0.42	0.00	0.11	15%	0%	4%	0.9289
3	0.66	0.00	0.15	20%	0%	5%	0.9258

**Table 13:** ANOVA model parameters for the load-bearing capacity of G1/G1 joints using factorial design.

Factorial Design G1/G1: 2 ^(2−0)^ Factor	ANOVA; Var.: Load-Bearing, kN; $R^{2}\;=\;0.92558;\;{\bar{R}}^{2}$ = 0.91699; MS Pure Error = 0.026482; DV: Load-Bearing, kN;				
	SS	df	MS	F	*p*
(1) Welding current setting, %	7.57870	1	7.578703	286.1835	0.000000
(2) Welding time, cycles	1.57038	1	1.570383	59.3001	0.000000
1 by 2	0.27230	1	0.272296	10.2823	0.003656
Lack of Fit	0.09551	1	0.095510	3.6066	0.069149
Pure Error	0.66205	25	0.026482		
Total SS	10.17894	29			

**Table 14:** Statistical indicators for assessing the quality and accuracy of fit for factorial design models.

Factorial Designs 2^(2-0)^	n	R^2^	${\bar{R}}^{2}$	r	MSE	RMSE	MAE	MAPE
Grade 1/Grade 1	30	0.92558	0.91699	0.9621	0.0253	0.1582	0.1337	5.3%
Grade 1/Grade 5	30	0.87264	0.85795	0.9342	0.0313	0.1711	0.1521	5.3%
Grade 5/Grade 5	30	0.87159	0.85677	0.9336	0.0654	0.2471	0.2133	6.5%

**Table 15:** ANOVA model parameters for the load-bearing capacity of G1/G1 joints using CCD.

Surface Design G1/G1 Factor	ANOVA; Var.: Load-Bearing, kN; $R^{2}\;=\;0.9682;\;{\bar{R}}^{2}$ = 0.96481; MS Pure Error = 0.0070525; 2 Factors, 1 Blocks, 53 Runs; DV: Load-Bearing, kN				
	SS	df	MS	F	*p*
(1) Welding current setting, % (L)	15.80437	1	15.80437	2240.968	0.000000
Welding current setting, % (Q)	0.04866	1	0.04866	6.899	0.012471
(2) Welding time, cycles (L)	3.51963	1	3.51963	499.064	0.000000
Welding time, cycles (Q)	0.01644	1	0.01644	2.331	0.135355
1 L by 2 L	0.28519	1	0.28519	40.439	0.000000
Lack of Fit	0.39691	10	0.03969	5.628	0.000046
Pure Error	0.26094	37	0.00705		
Total SS	20.68553	52			

**Table 16:** Statistical indicators for assessing the quality and accuracy of fit for CCDs.

Surface Design	n	k	R^2^	${\bar{R}}^{2}$	r	MSE	RMSE	MAE	MAPE
G1/G1	53	5	0.9682	0.96481	0.9840	0.0124	0.1118	0.0855	3.5%
G1/G5	52	5	0.9412	0.93485	0.9702	0.0173	0.1302	0.1016	3.7%
G5/G5	54	5	0.9517	0.94663	0.9755	0.0264	0.1616	0.1321	4.0%

**Table 17:** Results for ANN variants.

ID	Topology	Join Type	R^2^	${\bar{R}}^{2}$	r	MSE	RMSE	MAE	MAPE
A1	2-4-1	G1/G1	0.88793	0.88107	0.9423	0.0463	0.1835	0.1725	7.0%
A1	2-4-1	G1/G5	0.91055	0.90496	0.9542	0.0284	0.1451	0.1306	5.0%
A1	2-4-1	G5/G5	0.89714	0.89097	0.9472	0.0570	0.2370	0.1774	5.2%
A2	2-8-1	G1/G1	0.85496	0.84608	0.9246	0.0571	0.2313	0.1887	7.7%
A2	2-8-1	G1/G5	0.91177	0.90626	0.9549	0.0261	0.1548	0.1217	4.7%
A2	2-8-1	G5/G5	0.88968	0.88306	0.9432	0.0607	0.2302	0.1920	5.7%
A3	2-16-1	G1/G1	0.90913	0.90357	0.9535	0.0361	0.1905	0.1553	6.6%
A3	2-16-1	G1/G5	0.91177	0.90626	0.9549	0.0261	0.1548	0.1217	4.7%
A3	2-16-1	G5/G5	0.90280	0.89697	0.9502	0.0540	0.2312	0.1833	5.5%
B1	4-4-1	G1/G1	0.91083	0.90537	0.9544	0.0349	0.1822	0.1568	6.6%
B1	4-4-1	G1/G5	0.89502	0.88846	0.9461	0.0338	0.1577	0.1421	5.4%
B1	4-4-1	G5/G5	0.81618	0.80515	0.9034	0.1078	0.2611	0.2461	7.3%
**B2**	**4-8-1**	**G1/G1**	**0.91237**	**0.90701**	**0.9552**	**0.0359**	**0.1883**	**0.1488**	**6.1%**
**B2**	**4-8-1**	**G1/G5**	**0.92885**	**0.92440**	**0.9638**	**0.0210**	**0.1401**	**0.1134**	**4.3%**
**B2**	**4-8-1**	**G5/G5**	**0.92577**	**0.92131**	**0.9622**	**0.0414**	**0.2018**	**0.1520**	**4.6%**
B3	4-16-1	G1/G1	0.92386	0.91920	0.9612	0.0301	0.1740	0.1342	5.4%
B3	4-16-1	G1/G5	0.92660	0.92201	0.9626	0.0217	0.1461	0.1155	4.4%
B3	4-16-1	G5/G5	0.90566	0.90000	0.9517	0.0531	0.2284	0.1863	5.7%

**Table 18:** Predictive performance of baseline neural network benchmark models.

ID	Capacity Range [kN]	Estimated R^2^	Mean MSE [kN^2^]	Mean RMSE [kN]	SD_MSE_
B2(G1/G1)	1.45–2.05	0.96	0.00565	0.0752	0.00078
B2(G1/G5)	1.80–2.60	0.91	0.05520	0.2349	0.00510
B2(G5/G5)	2.26–3.06	0.94	0.01220	0.1102	0.00170

**Table 19:** Predictive performance of the B2 models under Grouped K-Fold validation.

ID	Number of Records	Number of Groups	Mean Trials per Group	Estimated R^2^	Mean MSE [kN^2^]	Mean RMSE [kN]	Mean SD_MSE_
B2(G1/G1)	53	16	~3.3	−4.32 *	0.0499	0.223	0.0242
B2(G1/G5)	52	16	~3.2	0.30	0.0540	0.232	0.0245
B2(G5/G5)	54	16	~3.4	0.24	0.0607	0.246	0.0268

* A negative R^2^ value indicates that, for the G1/G1 dataset, the variance of the target variable is sufficiently low that even small residuals lead to deterioration of the coefficient of determination.

**Table 20:** Parameters of the best individual.

Welding Current Setting [%]	Measured Current Intensity [kA]	Welding Time [Cycles]	Welding Time [s]	Load-Bearing [kN]
32	2.89	5.0	0.1	3.2

## Data Availability

The original contributions presented in this study are included in the article. Further inquiries can be directed to the corresponding author.
